# Designing Interventions that Last: A Classification of Environmental Behaviors in Relation to the Activities, Costs, and Effort Involved for Adoption and Maintenance

**DOI:** 10.3389/fpsyg.2017.01874

**Published:** 2017-11-06

**Authors:** Harriet E. Moore, Jennifer Boldero

**Affiliations:** ^1^Department of Geography, University of Melbourne, Melbourne, VIC, Australia; ^2^Department of Psychology, University of Melbourne, Melbourne, VIC, Australia

**Keywords:** environmental behavior, adoption, maintenance, designing interventions

## Abstract

Policy makers draw on behavioral research to design interventions that promote the voluntary adoption of environmental behavior in societies. Many environmental behaviors will only be effective if they are maintained over the long-term. In the context of climate change and concerns about future water security, behaviors that involve reducing energy consumption and improving water quality must be continued indefinitely to mitigate global warming and preserve scarce resources. Previous reviews of environmental behavior have focused exclusively on factors related to adoption. This review investigates the factors that influence both adoption and maintenance, and presents a classification of environmental behaviors in terms of the activities, costs, and effort required for both adoption and maintenance. Three categories of behavior are suggested. One-off behaviors involve performing an activity once, such as purchasing an energy efficient washing machine, or signing a petition. Continuous behaviors involve the performance of the same set of behaviors for adoption and for maintenance, such as curbside recycling. Dynamic behaviors involve the performance of different behaviors for adoption and maintenance, such as revegetation. Behaviors can also be classified into four categories related to cost and effort: those that involve little cost and effort for adoption and maintenance, those that involve moderate cost and effort for adoption and maintenance, those that involve a high cost or effort for adoption and less for maintenance, and those that involve less cost or effort for adoption and a higher amount for maintenance. In order to design interventions that last, policy makers should consider the factors that influence the maintenance as well as the adoption of environmental behaviors.

## Introduction

Policy makers draw on environmental psychology research to design interventions that promote environmental behavior in societies (Burton, 2004; Cialdini, 2007). The key purpose of these interventions is to motivate voluntary behavior change. This approach is an alternative to using traditional regulatory mechanisms to alter the way people behave in societies (Gunningham and Young, 1997). Behavioral research emphasizes the importance of tailoring interventions to address the barriers and facilitators of different behaviors (e.g., Michie et al., 2008, 2011; Osbaldiston and Schott, 2012).

Over the past decade a central focus of environmental policy worldwide has been responding to the threat of climate change. In the context of climate change, the biggest challenges faced by contemporary societies include reducing energy consumption, switching to renewable energy resources, and sustainably managing scarce resources, such as water in drought-prone countries. Reducing domestic consumption of energy and water is one way to of meeting these challenges. In 2004, 27% of total carbon emissions in the United Kingdom was the result of domestic consumption (Druckman and Jackson, 2008), while in the United States, the comparable figure for 2005 was 38% (Dietz et al., 2009). The importance of reducing these levels is reflected in the large body of research that investigates factors that influence behaviors such as reducing household temperature (Martinsson et al., 2011). Less attention has been paid to agricultural environmental behaviors associated with water quality. At the height of the recent decade-long drought in Australia (2009-2010) the agricultural sector was the largest consumer of water, consuming 52% of total water, followed by the domestic sector, consuming 14% of total water (ABS, 2012). In America (Agouridis et al., 2005) and Australia (Brooks and Lake, 2007), cattle grazing is one of the biggest contributors to declining water quality and the degradation or riverine ecosystems.

Addressing some of these challenges involves promoting behaviors that are relatively straight-forward and easy to perform, such as reducing energy consumption domestically. Others involve more complex, and costly behaviors, such as conservation in rural areas to improve water quality. To be effective, many environmental behaviors must be maintained indefinitely. However, most behavioral research examines factors that influence adoption only, and do not consider the factors influencing their maintenance over the long-term. In this paper we review theories about the factors that influence people to adopt pro-environmental behavior, and present an analysis about how environmental behaviors vary in terms of the barriers and determinants of adoption and maintenance. Osbaldiston and Schott (2012) observed that the design of an intervention to promote the adoption of environmental behaviors should match the amount of effort required to perform the new behavior. Further to this, we suggest that interventions are more likely to be effective if they are tailored to address the barriers and determinants of specific behaviors not only at the time of adoption, but also over the long-term.

According to Rothman (2000), behaviors can fail to be maintained, even when adoption has been successful because, “the decision criteria that lead people to initiate change in their behavior are different from those that lead them to maintain it” (p. 64). The discontinuation of newly adopted behaviors is a risk if maintenance involves additional activities that pose new challenges and barriers (Rothman, 2000). The problem of discontinuation has received little attention in environmental behavioral literature. By comparison, discontinuation is addressed comprehensively in health literature in relation to facilitating participation in exercise regimes (Akers et al., 2010), the cessation of smoking (Prochaska et al., 1991; Cahill and Perera, 2008), and weight loss (Jeffery et al., 2000). For example, the PRECEDE-PROCEED model of health behavior proposes that behavior adoption and maintenance are determined by different factors (Grol and Wensing, 2004). An intention to adopt a behavior is related to “predisposing factors,” such as attitudes. Performance is related to “enabling factors,” such as capacity and resources, and “reinforcing factors,” such as social norms. Thus, it is necessary to identify the specific factors related to adoption and maintenance to design effective interventions. This principle is equally as important for the design of interventions that promote environmental behavior.

We suggest that some behaviors may be more prone to failure than others. Identifying behaviors that are more likely to face barriers, and thus the risk of failure, is critical to the success of environmental endeavors to mitigate climate change and protect scarce resources. For this purpose we reviewed 56 environmental psychology papers, and identified 39 unique environmental behaviors. In the following we present four observations that will assist policy makers with designing interventions that promote pro-environmental behaviors, and researchers with designing studies that capture the full range of factors that influence behavior.

Our first observation is that there is no single theoretical approach that explains all instances of environmental behavior, rather, some approaches explain specific *types* of behavior better than others. Behaviors that face few, if any, practical barrier are likely to be related to cognitive factors, such as attitudes and moral norms (Steg, 2008). In contrast, behaviors that face barriers such as cost, are likely to be related to both cognitive factors such as attitudes, and practical factors, such as cost and effort (Guagnano et al., 1995). Therefore, theories that examine both cognitive and practical factors, such as the Theory of Planned Behavior (TPB) (Ajzen, 1985, 1991), are more appropriate for researching behaviors that face barriers. Further, models that include past behavior are more likely to explain environmental behaviors that are relatively easy to perform, involve consistent activities over time, and are repeated frequently, compared to behaviors that occur irregularly (Verplanken and Aarts, 1999).

Our second observation is that although the environmental research literature refers to “behavior change,” most target “behaviors” involve the performance of multiple activities, often over long periods of time rather than a single activity, or a single instance. Other researchers group behaviors in terms of similar goals or purposes (e.g., Kaiser and Wilson, 2004). We classify behaviors into three categories in terms of the activities involved for adoption and maintenance. The first category is behaviors that involve one-off activities and little to no maintenance, such as purchasing an energy-efficient washing machine. The second category is behaviors that involve the same activities at the time of adoption as over the long-term, such as curbside recycling. These we term “continuous” behaviors. The third category is behaviors that involve different activities for adoption and continued maintenance, such as the revegetation of riverbanks. Adoption of revegetation projects involves planting saplings, while maintenance involves extensive weed management. These we term “dynamic” behaviors.

Our third observation is that *some environmental behaviors are more difficult to adopt and maintain than others* because they face practical barriers, such as cost and effort. Typically altering existing behavior involves less cost and effort than performing entirely new activities (Binder and Boldero, 2012). Domestic and daily life behaviors, such as recycling and travel, tend to involve altering existing behavior. These behaviors involve few costs and little effort. Most agricultural environmental behaviors involve performing entirely new activities, such as building fences to keep cattle from accessing riverbanks. These behaviors tend to involve high costs and high degrees of effort. Therefore, agricultural behaviors pose a greater challenge to policy makers than those behaviors that are performed in daily life.

Our final observation is that *interventions to promote pro-environmental behavior should be tailored to the activities and determinants involved for both the adoption and the maintenance of behaviors*. Interventions that appeal to attitudes and social norms through education campaigns are more suitable for promoting behaviors that are low-cost and easy to perform. Interventions that appeal to moral norms are more likely to be effective for promoting behaviors that are low-cost, but involve a greater degree of effort. Interventions that involve subsidies are more effective for promoting behaviors that involve high-costs. Further, behaviors that involve different activities for adoption and maintenance are more likely to require additional incentives than those that involve the same activities over the long-term.

Others have reviewed the literature to determine the factors associated with the adoption of environmental behavior (e.g., Bamberg and Möser, 2007; Steg and Vlek, 2009). For example, Steg and Vlek (2009) analyzed the factors that determine behavior, and classified interventions as either educational or structural. Further, Michie et al. (2011) argued that the determinants of different types of behavior must be considered if interventions are to be effective. In this paper we:

- Outline common theoretical perspectives;
- Review literature about environmental behavior and emphasize knowledge and knowledge gaps about the maintenance of new behaviors, in addition to the initial adoption of behaviors;
- Present a classification of behaviors in terms of whether they are one-off, continuous, or dynamic, and the cost and effort associated with adoption and maintenance; and
- Put forward some recommendations for research and the design of interventions.

To mitigate environmental problems, such as catastrophic climate change, societies need adopt and maintain pro-environmental behaviors. In order to design effective interventions, researchers and policy makers must first understand the dynamics of those behaviors.

## Literature review

To determine commonly used behavioral models and common environmental behaviors, we conducted a literature review of peer reviewed environmental behavior papers from online databases, including papers that present the results of experimental studies, natural studies, and meta-reviews. Two strategies were used to identify relevant studies. The first strategy was to enter search terms into data bases, including Google Scholar, and Web of Science. The second strategy was to examine the reference list of studies that were identified through data base searches. This method was chosen over the examination of journal contents for two reasons; firstly, searching references can yield a greater amount of unique studies, and secondly, this method is more efficient in terms of the time required to identify unique studies (Greenhalgh and Peacock, 2005). These two strategies were employed reflexively such that new search terms were identified through the examination of reference lists.

Initially, broad search terms were used to identify a wide range of environmental behaviors, including the following: environmental behavior, pro-environmental behavior, environmentally friendly behavior, conservation behavior, restoration behavior, domestic environmental behavior, rural environmental behavior, environmental behavior, and the Theory of Planned Behavior, environmental behavior and morals, environmental behavior and habits, environmental behavior and adoption, environmental behavior and maintenance, environmental behavior and continuation, and environmental behavior and social norms. These search terms yielded a range of studies, including meta-reviews (e.g., Bamberg and Möser, 2007) that were useful for identifying bodies of literature about specific types of environmental behavior, such as traveling behavior and consumer purchasing behavior.

A second more extensive search was conducted using terms related to specific environmental behaviors, including the following: travel mode choice, car use, public transport use, cycling, purchasing electric cars, purchasing electric bikes, purchasing alternative fuel cars, bus use, choosing to walk, domestic water conservation, domestic energy conservation, recycling, separating waste, waste management, curb-side recycling, drop-off recycling, composting, installing blinds, installing insulation, purchasing green products, purchasing energy saver white goods, agricultural environmental behavior, agricultural water conservation, river restoration behavior, stock exclusion, revegetation, activism, attending rallies, non-violent direct action, signing petitions, environmental political actions, organic food, vegetarianism, eating meat, local produce, installing water tanks, and installing solar panels.

The identified studies were coded in two ways. The first level of coding was to identify the specific behavior or behaviors examined in the study, such as curb-side recycling or choosing to take public transport. The second level of coding was to identify the theoretical model, such as the TPB, or individual factors, such as attitudes and social norms, that were used to examine environmental behaviors.

Of 189 peer reviewed studies identified, 78 were selected on the basis of three criteria. Importantly, in contrast to previous reviews, the purpose of our review was to identify the specific determinants of individual behaviors. Most reviews lump multiple behaviors or multiple determinants together. Thus, our criteria were selected to fulfill this purpose. The first criterion was studies that either used an established psychological theory of behavior change, such as the TPB (Ajzen, 1985, 1991), or used psychological methods to investigate the relationship between a single psychological construct, such as social norms, and environmental behavior. The second criterion was studies that made explicit the environmental behaviors and activities under investigation, and reported bivariate results for the relationship between individual behaviors and determinants. Unlike Bamberg and Möser (2007) we included studies that used multivariate analysis to examine the relationship between multiple determinants of a single behavior. We excluded studies that referred to environmental behaviors without specifying the activities that were measured or studies that collapsed multiple related activities into a single aggregate variable (e.g., Grob, 1995). This was necessary because our analysis considers the specific activities involved for individual behaviors. Some behaviors involve the same activities, and therefore may be related to the same determinants, such as purchasing eco-friendly detergents and purchasing recycled toilet paper. However, this is not always the case. Many environmental behaviors involve very different activities, regardless of whether the end goal is similar. People behave inconsistently; someone who recycles may not necessarily practice water conservation (Steg and Vlek, 2009). Even within a single domain, such as household energy saving, specific behaviors may have very different determinants (Abrahamse and Steg, 2009). For example, Tonglet et al. (2004) surveyed households about waste management and found that while 80% recycle, only 40% purchase with the aim of reducing waste, and 55% repair or reuse items to reduce waste. These behaviors involve vastly different activities, and, if considered as a single measure of “waste management,” would not reflect the prevalence of each behavior in society, or the determinants related to specific activities.

We also excluded studies that lacked specificity. In particular, numerous studies fail to distinguish between curbside, central location, and public recycling (Osbaldiston and Schott, 2012). The lack of specificity is problematic because each type of recycling involves different activities, and, “there are substantial differences in the forethought and effort required to perform [the] different behaviors” (Osbaldiston and Schott, 2012, p. 280). For example, Ebreo et al. (1999) treated curbside and drop-off recycling as a single behavior and used a single measure of environmental attitudes toward recycling to examine the relationship between attitudes and behavior. Ajzen (1996) suggests that attitudes and behaviors should be measured at the same level of specificity. The lack of specificity between the measure and the behaviors reduces the reliability of the study findings. Similarly, Beedell and Rehman (1999) analyzed semi-structured interview data from farmers in Bedfordshire to investigate the relationship between the factors of the TPB, and conservation behavior. However, the study does not distinguish between the multiple activities involved in conservation, such as weed management and tree-planting.

The third criterion was that the study or review investigated the relationship between determining factors and either behavior intentions, self-reported behavior, or observations of actual behavior. For example, studies that explored the relationship between attitudes toward different environmental issues without examining links to behavior were excluded (e.g., Larsen, 1995). In the reference section of this paper the studies included in our literature review are marked with an asterisk. Although not exhaustive, the studies included in our review extend the range of environmental behaviors that were the focus of earlier reviews, including those of Bamberg and Möser (2007), Osbaldiston and Schott (2012), and Steg and Vlek (2009). We recognize that a variety of other theoretical approaches, not included in this review, are also used to study environmental behavior. However, for the purposes of this paper we highlight the most common approaches (Bamberg and Möser, 2007); models that focus on factors related to altruism, such as Schwartz (1977) model, and models that focus on factors related to self-interest, such as rational choice models (e.g., Ajzen, 1985, 1991), and additionally models about automated behavior (e.g., Verplanken and Aarts, 1999).

The findings of the literature review are presented in the next two main section: the first on theoretical perspectives, and the second on common environmental behaviors and their behavioral determinates.

## Theoretical perspectives

Environmental behaviors vary in terms of the activities, costs, and efforts involved for adoption and maintenance. As a result, the barriers and determinants of behavior also vary (Michie et al., 2011). Therefore, individual theories of behavior change are better suited to some instance of environmental behavior than others. In the following we summarize the most common behavioral theories that are used to examine environmental behavior.

### Models based on altruism

The main proposition of altruistic models of environmental behavior is that social moral obligations extend to environmental responsibility (Stern, 2000). The Norm Activation Model (NAM) (Schwartz, 1977) suggests that altruism is the result of an internal sense of right and wrong (i.e., personal norms) that produce guilt when moral obligations are not fulfilled, and self-esteem when they are fulfilled. Thus, the performance of environmental behavior is the outcome of self-reinforcement rather than social reinforcement. Moral norms are activated when individuals become aware of adverse consequences of behavior and take responsibility for those consequences (Schwartz, 1977). An extended Value-Belief-Norm model (VBN) proposed by Stern and Dietz (1994) suggested that personal, social, and biospheric consequences influence the process of norm activation.

Of the studies included in our review, 10 considered the relationship between moral norms and the performance of pro-environmental behavior. However, considerably more considered the role of moral norms in conjunction with other factors, such as attitudes. These approaches are outlined below in the discussion of integrated models. Together, models of altruism and models of altruism in combination with other variables were used to examine environmental behaviors from across all the six categories of behavior identified in our review: transport, household consumption, purchasing, waste management, agriculture, and miscellaneous.

### Models of goal direction

Models that assume environmental behavior is goal-directed include those that focus on intentional, rational choice, and those that focus on habitual behaviors. Both perspectives suggest that pro-environmental behavior is driven by goal-directed self-interest, however the degree of conscious decision-making varies. Models of intentional behavior suggest that rational decision-making is involved for every instance of performing a behavior (e.g., Ajzen, 1985, 1991). Models of habitual behavior suggest that while conscious intent is initially involved for the performance of behavior (Klöckner, 2013), the frequency of repetition, ease of performance, and consistency of context can result in automaticity, and therefore the formation of habits (Verplanken and Aarts, 1999). Both perspectives on behavior driven by self-interest are considered in the following.

#### Models of intentional behavior

Models of intentional behavior suggest that the performance of environmental behavior is the result of the rational consideration of cognitive factors, like attitudes, beliefs, and norms, and external factors, such as cost and time. While numerous rational choice models have been put forward (e.g., Rogers, 1975; Keshavarz and Karami, 2016), the most common used to research environmental behavior (Klöckner, 2013) are the Theory of Reasoned Action (TRA) (Ajzen and Fishbein, 1980; Fishbein and Ajzen, 1981), and its extension, the TPB (Ajzen, 1985, 1991). These models suggest that most human behavior is goal-directed, and therefore intentional. The TPB proposes that intentions to engage in a behavior are influenced by attitudes, social norms, and perceived behavioral control (PBC). Ajzen (1991) argues that PBC exerts influence on behavior by way of intentions, and directly. PBC includes factors related to self-efficacy, or external practical factors (Armitage and Conner, 2001).

Of the studies included in our review 39 examined the relationship between TPB, TRA, or individual factors included in these models, and the performance of environmental behavior. Numerous others combined these models with other variables, including moral norms and habits, as reported below in relation to integrated approaches. Models of intentional behavior were found to explain examples of behavior from across the six categories of environmental behavior examined in our review.

#### Models of habitual behavior

Habits refer to the “automatic performance of behavioral patterns triggered by context cues” Klöckner (2013, p. 1030). Many habits serve a function and therefore involve intentionality (Verplanken and Aarts, 1999). Three conditions determine habit formation: the degree of involvement, perceived complexity, and the degree of constraints (Jackson, 2005). Behaviors that are easier to perform, are performed more regularly, and face fewer constraints are more likely to become habitual (Bratt et al., 2015). For example, curbside recycling (e.g., Bratt, 1999; Osbaldiston and Schott, 2012) and water saving (Martínez-Espiñeira et al., 2014) are generally considered easy to perform and face few, if any barriers. In both cases, past behavior has been found to predict behavioral intentions (Carrus et al., 2008; Martínez-Espiñeira et al., 2014). Further, the determinants of habits can vary depending on the strength of the habit. Strong habits are triggered predominately by context rather than intentions and goals, while weaker habits are more directly influenced by goals (Neal et al., 2012). Habit strength is determined by the frequency and consistency of past performance (Ouellette and Wood, 1998; Aarts and Dijksterhuis, 2000). While behaviors become automatic over time, habitual behavior often begins with intentions (Verplanken and Aarts, 1999). Over the time the focus of the behavior shifts from performing specific activities, to the goal of performance, as the sequence of activities becomes routine (Thøgersen, 1999).

Of the studies included in our review six considered the influence of past behavior on the performance of pro-environmental behavior. Interestingly, we found that the role of habits has been considered for each type of environmental behavior outlined below, except for agricultural environmental behavior. It is of course possible that we have overlooked some studies of this nature. However, it may be that agricultural environmental behaviors involve complex activities that are unlikely to become habitual. Our discussion of the activities involved in common agricultural environmental behaviors below suggests that this may be at least a partial explanation.

### Integrated models

Numerous scholars have integrated variables and concepts from multiple theoretical models to conduct research about environmental behaviors (e.g., Kaplan, 2000; Stern, 2000; Corbett, 2005). Of the studies included in our review 13 combined elements of multiple theories or models. The most common approaches were the integration of factors associated with the TPB and moral norms (Harland et al., 1999; Davies et al., 2002; Valle et al., 2005; Chen and Chai, 2010; Chan and Bishop, 2013; Chen and Tung, 2014; Botetzagias et al., 2015; Yazdanpanah and Forouzani, 2015). Many others integrated the TPB with past behavior (e.g., Hamid and Cheng, 1995; Bamberg and Schmidt, 2003).

The popularity of integrated models reflects the growing recognition that any one model is insufficient for explaining all instances of environmental behavior (e.g., Michie et al., 2011). For example, the TPB is often critiqued for failing to consider morality and past behavior (Klöckner, 2013). To address this critique, Klöckner (2013) proposed the Comprehensive Action Determination Model (CADM) of environmental behavior that integrates key concepts from the four most common theories of environmental behavior: TPB, VBN, NAT, and theories about habitual behavior. In particular, the CADM addresses the fact that, “Typically the TPB and the NAT/VBN tradition perform notoriously poorly for repeated behaviors.” (Klöckner, 2013, p. 1030). This observation is relevant to our analysis of environmental behaviors as we distinguish between the activities and determinants associated with the initial adoption of a new behavior, and the activities and determinants associated with the continued maintenance of a new behavior. We argue that in some instances, the activities and determinants are stable over time, while in other instances they are different for adoption and maintenance.

Klöckner (2013) suggested that the TPB, VBN/NAT, and theories about habits are complementary in the following manner. Firstly, it is now widely accepted that the inclusion of moral norms increases the predictive power of TPB. Moral norms are an important predictor of intention (Klöckner, 2013). Secondly, it is likely that habit strength moderates the relationship between intention and behavior. Intentions are a stronger predictor of behaviors that are only rarely performed, while past behavior is a stronger predictor of behaviors that are performed regularly (Ouellette and Wood, 1998). Therefore, Klöckner (2013) argued that, “At an earlier point in time, when a behavior was performed for the first couple of times, intentions and PBC were the main determinants. By repeating it, a habit was established and it took over control from the two variables.” (p. 1031). For example, attitudes may influence the adoption of new curbside recycling behavior (Vining and Ebreo, 1992) or the initial shift to household energy use reduction (Abrahamse and Steg, 2009), while past behavior may be a stronger predictor of behavior than attitudes 6 months after initial adoption. In summary, integrated models of environmental behavior combine elements of the most common theoretical approaches.

### Summary of theoretical perspectives

Common theoretical models suggest that environmental behavior is influenced by factors related to altruism and goal directed intentional self-interest, however it is widely accepted that morality is an important contributor to intentions. Goal directed behavior varies according to the degree to which it is intentional and conscious or habitual and automatic. Factors related to altruism, such as morality, and factors related self-interest, such as attitudes, can influence the initial performance of a new environmental behavior. Some environmental behaviors are likely to become habitual, while others may involve intentional decision-making on every occasion. Over time, past behavior is more likely to explain environmental behaviors that are easy to perform and are performed frequently, such as curbside recycling. By contrast, environmental behaviors that are difficult or costly to perform and are performed infrequently, such as purchasing energy efficient white goods, are more likely to be explained by factors related to intention.

In the following we examine the characteristics of common environmental behaviors, including common determinants of different behaviors, and the activities involved for the adoption and maintenance of new behaviors.

## Characteristics of environmental behavior

Environmental behaviors vary in terms of the activities involved, and the determinants that influence adoption and maintenance. In the following we summarize the determinants of six common categories of behaviors, and classify these behaviors as either “one-off,” “continuous,” or “dynamic” on the basis of the activities involved for adoption and maintenance. Further, we consider the cost and effort required to perform the adoption and maintenance of behaviors, and discuss the implications for designing interventions. Table 1 summarizes our classification of behaviors in terms of the activities involved for adoption and maintenance, common behavioral determinants, and the effort and cost involved for performing the environmental behaviors considered in this review.

**Table 1:** Classification of environmental behaviors in terms of actions, costs, and effort required for adoption and maintenance.

		**Environmental behavior**	**Adoption activities**	**Maintenance activities**	**Cost/Effort category**	**Determinants**	**Examples of studies**
Transport	Continuous	Curtailing car use	Replacing traditional activities with new activities	Continuation of new activities	L/L	Personal norms	Nordlund and Garvill, 2002
						TPB and personal norms	Harland et al., 1999; Corbett, 2005
		Taking public transport	Replacing traditional activities with new activities	Continuation of new activities	L/L	Habits	Carrus et al., 2008
						Personal norms	Bamberg et al., 2007
		Travel mode choice (e.g., car, bike, public transport)	Replacing traditional activities with new activities	Continuation of new activities	L/L	Attitudes	Nilsson and Küller, 2000
						Habits	Aarts and Dijksterhuis, 2000
						Habits and personal norms	Klöckner and Matthies, 2004
						TPB and habits	Bamberg and Schmidt, 2003
	Dynamic	Switching to an electric car	Purchasing car and home charging station	Ongoing use, maintenance and charging battery	H/L	Protection Motivation Theory	Bockarjova and Steg, 2014
						TPB	Wang et al., 2016
						Financial factors	Graham-Rowe et al., 2012; Barth et al., 2016
						Attitudes	Egbue and Long, 2012; Barbarossa et al., 2015
						Emotions and symbolism	Moons and De Pelsmacker, 2012;
		Switching to a hybrid car	Purchasing	Ongoing use and maintenance	H/L	Financial factors	Ozaki and Sevastyanova, 2011
						Values, education, financial factors	Carley et al., 2013
		Switching to an alternative fuel car	Purchasing	Ongoing use, maintenance and supply of alternative fuel	H/L	Education, values, beliefs, and personal norms	Jansson et al., 2011
		Switching to an electric bicycle	Purchasing or retrofitting with kit	Ongoing use, maintenance, and charging battery	M/M	Attitudes and norms	Wolf and Seebauer, 2014; Kaplan et al., 2015
Household consumption	One-off activities	Installing double glazing/insulation	Installing and purchasing technology	N/A	H/L	Financial factors	Martínez-Espiñeira et al., 2014
		Purchasing energy efficient white goods	Installing and purchasing technology	Continuing to use technology.	H/L		
		Replacing showerhead	Purchasing and installing technology.	Continuing to use technology.	H/L	Cognitive factors	Steg, 2008
		Switching to green energy company	Organizing transfer and associated fees	Paying more for electricity	M/M	Knowledge	Pichert and Katsikopoulos, 2008
						Attitudes, knowledge, and financial factors	Arkesteijn and Oerlemans, 2005
		Installing wood pellet heating system	Installing and purchasing technology	Continuing to use technology	H/L	TPB	Sopha and Klöckner, 2011
		Using smart energy system	Installing system	Continuing to use system	H/L	Symbolic values	Noppers et al., 2016
	Continuous	Curtailing energy or water use	Replacing traditional activities with new activities involved for pro-environmental behavior	Continuation of new activities	L/L	Habits	Maréchal, 2009; Martínez-Espiñeira et al., 2014
						PBC and personal norms	Abrahamse and Steg, 2009
						Attitudes, beliefs, norms	Black et al., 1985
						Habits	Gregory and Leo, 2003
		Using energy saver light bulbs				Personal norms	Thøgersen, 2006
		Reducing household temperature/hot water use				Attitudes and income	Martinsson et al., 2011
		Doing only full dishwasher or washing machine loads/filling sink for doing dishes				Habits	Martínez-Espiñeira et al., 2014
		Turning off faucet for teeth brushing				TPB and moral norms	Harland et al., 1999
	Dynamic	Switching to solar electricity	Installing and purchasing technology	Ongoing maintenance and paying less for electricity	H/L	Financial factors	Claudy et al., 2013; Schelly, 2014
						TPB and financial factors	Korcaj et al., 2015
Purchasing	Continuous	Eco-cleaning products Fair trade Organic Amount of meat Sustainable meat Unbleached toilet paper Recycled toilet paper Eco-friendly packaging Bringing containers for purchasing Taking plastic bags when offered Not purchasing products tested on animals	Substituting traditional products or purchasing behavior for environmentally friendly alternatives	Continuing to perform environmentally friendly purchasing behavior	L/L	Attitudes	Tanner and Wölfing Kast, 2003; Fraj and Martinez, 2006; Liang, 2016
						Habits and effort	Dahab et al., 1995
						TPB and personal norms	Chen and Chai, 2010; Yazdanpanah and Forouzani, 2015
						Personal norms	Dietz et al., 1995; Thøgersen, 1999
Waste management	Continuous	Curb-side recycling	Separating waste, storing recycling, depositing recycling in container on curbside	Separating waste, storing recycling, depositing recycling in container on curbside	L/L	TPB	Tonglet et al., 2004
						TPB and personal norms	Botetzagias et al., 2015
						Attitudes	Vicente and Reis, 2008
						Socio-economic factors	Martin et al., 2006
						Habits	Carrus et al., 2008
						Personal norms	Allen and Ferrand, 1999;
						Personal norms and PBC	Davies et al., 2002
		Drop-off recycling	Separating waste, storing recycling, traveling to drop-off center/paying a fee	Separating waste, storing recycling, traveling to drop-off center/paying a fee	M/M	Cost	Guagnano et al., 1995
						Personal norms	Bratt, 1999
						Attitudes	Schultz and Oskamp, 1996
		Composting	Separating and storing organic waste	Separating and storing organic waste	L/L	TPB	Taylor and Todd, 1995
						Personal norms	Thøgersen, 2006
Agriculture	One-off	Water saving technology	Initial cost of installation	Continuing to use technology	H/L	PBC	Lynne et al., 1995
	Continuous	Reduce tillage	Change tillage practices	Continue new practices	L/L	Attitudes	Wauters et al., 2010
	Dynamic	Plant cover crops/buffers strips	Planting seedlings	Replacing lost seedlings and weeding	L/H	Attitudes	Wauters et al., 2010
		Improved vegetation management	Fertilizer use, plant new species	Continued fertilizer use, weed management	L/H	Attitudes, norms and PBC	Borges et al., 2014
						VBN and place attachment	Raymond et al., 2011
Miscell	One-off	Signing an anti-pollution petition	Signing petition	N/A	L/L	TRA and past behavior	Hamid and Cheng, 1995
	Continuous	Visiting “green” hotels	Choosing green hotels	Choosing green hotels	L/L	TPB and moral obligation	Chen and Tung, 2014

^*^ Table contains examples of studies included in our review. The full selection is contained in the reference section.

^**^ L/L, little if any cost, and minimal effort required for both adoption and maintenance; M/M, moderate cost or effort required for adoption and maintenance; H/L, high up-front cost for adoption, and either no cost for maintenance or less cost than the conventional alternative; L/H, low to moderate cost for adoption and a higher cost for maintenance.

### Common environmental behaviors and determinants

Through our review of 78 studies we identified 46 environmental behaviors, which fall into six categories: transportation, household consumption, purchasing, waste management, agriculture, and miscellaneous (see Table 1). We acknowledge that there are many additional environmental behaviors that are not included in our review, primarily because many studies did not meet our selection criteria.

#### Transportation

Of 78 papers reviewed, 26 examined the determinants that influence environmental behaviors related to transport choice, particularly curtailing car use, choosing to use other modes of transport compared to car use, and the purchase and use of electric, hybrid, and alternative fuel vehicles. Some studies suggest that travel choice is habitual (Aarts and Dijksterhuis, 2000; Carrus et al., 2008). Further, it is possible to alter travel habits through intervention, such as by subsidizing public transport use (Hunecke et al., 2001), or increasing ease of access to public transport (Bamberg et al., 2003).

Other studies indicate that factors related to self-determination, such as attitudes (e.g., Nilsson and Küller, 2000), and altruism, such as personal norms (e.g., Nordlund and Garvill, 2002; Klöckner and Matthies, 2004; Thøgersen, 2006; Bamberg et al., 2007), explain travel choice. In addition, multiple studies have found that travel choice is explained by the TPB both singularly (e.g., Bamberg et al., 2003) and combined with other factors, such as personal norms (Harland et al., 1999), and habits (Bamberg and Schmidt, 2003).

Numerous factors are related to the adoption and use of electric, hybrid, and alternative fuel vehicles, such as attitudes (Egbue and Long, 2012; Barbarossa et al., 2015; Wang et al., 2016), self-efficacy (Bockarjova and Steg, 2014; Langbroek et al., 2016), moral norms (e.g., Jansson et al., 2011), values (e.g., Carley et al., 2013), and emotions (e.g., Moons and De Pelsmacker, 2012). However, the most common determinants are factors related to cost (e.g., Flamm and Agrawal, 2012; Hahnel et al., 2014; Lai et al., 2015; Barth et al., 2016).

Multiple studies found that the high purchasing cost is a barrier to adoption, while lower use costs compared to conventional vehicles, is a driver of adoption (e.g., Ozaki and Sevastyanova, 2011; Graham-Rowe et al., 2012). Further, pro-environmental values and attitudes, and social norms, are less influential than financial considerations (Hahnel et al., 2014; Barth et al., 2016). For example, Mairesse et al. (2012) observed that, “most people have positive attitudes towards the environment, but they do not prevail over attitudes associated with other car attributes” (p. 568), such as cost.

In comparison to the adoption and use of sustainable cars, the adoption of electric bicycles is positively related to attitudes and norms toward technology, and negatively related to norms about physical activity (Wolf and Seebauer, 2014; Kaplan et al., 2015). Taken together, these findings suggest that a number of factors can influence initial decisions about travel choices that are relatively inexpensive, and without substantive barriers habits may form around those decisions. These travel habits may also be altered through interventions. However, the adoption of behaviors that involve purchasing expensive sustainable technologies, such as electric cars, is influenced substantially by factors related to cost.

#### Household consumption

Twelve studies included in our review examined the determinants that influence environmental behaviors related to household consumption. Most studies consider multiple purchasing behaviors. Unlike most other environmental behaviors, the purchasing behaviors considered here involve the same activities: switching from conventional items to environmentally friendly items.

Both cognitive and practical factors influence the adoption of pro-environmental household consumption behaviors. However, the degree that each factor influences behavior depends both on the nature of the behavior and the context (Black et al., 1985). Easy behaviors are more likely to be related to cognitive factors, such as attitudes, while difficult behaviors are more likely to be related to practical factors, such as socio-economic context. For example, personal norms were found to be related to behaviors that face few barriers, such as reducing energy consumption (Abrahamse and Steg, 2009) and using energy saver lightbulbs (Thøgersen, 2006).

Both cognitive factors and financial factors were found to influence the adoption of green power (Arkesteijn and Oerlemans, 2005; Pichert and Katsikopoulos, 2008; Ozaki, 2011), and the installation of wood pellet heating systems (Sopha and Klöckner, 2011), smart energy systems (e.g., Noppers et al., 2016), and solar power systems (Claudy et al., 2013; Schelly, 2014; Korcaj et al., 2015). This is not surprizing given that green power typically costs substantially more than traditional power (Roe et al., 2001) and wood pellet heating and solar systems are expensive to install (Sopha and Klöckner, 2011; Schelly, 2014). Similarly, studies have found that simple water saving activities, such as turning off the faucet for teeth brushing and filling the washing machine before doing a load, are related to moral norms (Harland et al., 1999) and habits (Gregory and Leo, 2003; Martínez-Espiñeira et al., 2014). By comparison, purchasing water saving technologies, such as a water efficient washing machine, is more greatly influenced by financial factors, such as income (Martínez-Espiñeira et al., 2014).

Research also suggests that context, such as financial insecurity, also prevent households from adopting pro-environmental consumption behaviors. For example, Martinsson et al. (2011) found that attitudes are more influential for energy saving behavior in high-income homes compared to low-income homes. In summary, cognitive, practical, and contextual factors influence pro-environmental household consumption behaviors. However, those behaviors that are easier to perform are more likely to be influenced by cognitive factors while those behaviors that are more difficult to perform, particularly if high costs are involved, are more likely to be influenced by practical factors. Following the above review of environmental behaviors related to transport, we might also expect that easier and frequent household behaviors, such as reducing energy consumption and turning off the faucet for teeth brushing, are more likely to result in the formation of habits (e.g., Gregory and Leo, 2003; Maréchal, 2009; Binder and Boldero, 2012).

#### Purchasing

Thirteen studies from our review explored the relationship between determinants and the performance of pro-environmental purchasing behavior. Pro-environmental purchasing behavior involves either substituting traditional products for environmental friendly products, or reducing consumption, such as reducing meat purchasing and avoiding packaging. Most purchasing behaviors are relatively simple to perform, and many, such as purchasing recycled toilet paper, are low-cost. However, some purchasing behaviors face moderate barriers such as cost, effort, access (Davies et al., 2002; Tanner and Wölfing Kast, 2003), and context (Liang, 2016). For example, Tanner and Wölfing Kast (2003) found that the time taken to access fair trade and local produce was a barrier to purchasing behavior. Further, Liang (2016) found that the relationship between attitudes and intention was stronger for instances of low-priced organic foods compared to high-priced organic foods.

Factors related to self-interest, including attitudes and social norms, are related to purchasing behavior including purchasing recycled paper, eco-detergents, organic produce, ozone-friendly products, and avoiding products tested on animals (e.g., Schlegelmilch et al., 1996; Bissonnette and Contento, 2001; Tanner and Wölfing Kast, 2003; Fraj and Martinez, 2006). Personal norms have also been found to be related to packaging waste prevention (Thøgersen, 1999), buying organic milk (Thøgersen, 2006), and choosing not to purchase meat (Dietz et al., 1995). Further, models that combine factors related to both self-determinism and altruism have found to explain a range of purchasing behaviors (Harland et al., 1999; Chen and Chai, 2010; Yazdanpanah and Forouzani, 2015).

Further, purchasing behaviors may also become habitual. Dahab et al. (1995) found that both habits and perceived effort predicted purchasing behavior. Perceived effort influences the initial trial of purchasing recycled materials, and perceptions of the initial trial influence how likely a behavior is to become habitual. In summary, the initial act of purchasing pro-environmental products can be motivated by a range of factors. How the initial trial of a new behavior is perceived may determine whether the behavior becomes habitual, and thus whether the behavior is likely to be maintained.

#### Waste management

Of 22 studies about pro-environmental waste management, all examined recycling behavior, and two examined composting behavior. Both the TPB (Taylor and Todd, 1995) and personal norms (Thøgersen, 2006) have been found to explain composting behavior.

The determinants of recycling vary depending on the activities involved and convenience. Common barriers include the cost of drop-off recycling, time, and limited storage space for items (e.g., De Young, 1990; Guagnano et al., 1995; Martin et al., 2006). Some studies suggest that curbside recycling behavior is explained by the TPB (e.g., Chan, 1998; Tonglet et al., 2004), and TPB with the inclusion of moral norms (Chan and Bishop, 2013; Botetzagias et al., 2015) and identity (Nigbur et al., 2010). Others have found that individual factors associated with rational choice, such as attitudes (e.g., De Young, 1990; Vining and Ebreo, 1992; Schultz et al., 1995; Schultz and Oskamp, 1996; Vicente and Reis, 2008), and PBC (e.g., Taylor and Todd, 1995) determine recycling when there are few barriers to practice. When recycling is inconvenient, such as in the case of poorly organized curbside recycling services, social-economic factors (Martin et al., 2006) and demographic factors (Lansana, 1992) are better predictors of behavior than cognitive factors like attitudes. It has also been suggested that the relationship between cognitive constructs and recycling behavior is multidirectional. For example Werner et al. (1995) found that engaging in recycling behavior strengthens positive attitudes toward recycling.

Multiple studies of recycling behaviors support Schwatz's norm activation model of altruistic behavior (e.g., Allen and Ferrand, 1999; Thøgersen, 1999), and Schwatz's model in combination with variables associated with the TPB such as PBC (e.g., Davies et al., 2002; Valle et al., 2005). Unlike factors related to rational choice, the relationship between altruism and recycling behavior is weaker when the activities involved are easy to perform (e.g., Bratt, 1999). For example, Guagnano et al. (1995) found that Schwatz's model only explained recycling in the absence of convenient curbside recycling services.

Past behavior, and therefore the formation of habits, also explains recycling when recycling activities are relatively easy to perform (Carrus et al., 2008). Similarly, González-Torre and Adenso-Díaz (2005) found that the frequency of recycling increased as distance from recycling facilities decreased. In summary, self-determination may influence the initial performance of waste management behavior in the absence of constraints, while altruism is likely to influence behavior in the presence of constraints. Habits form when behavior is easily repeated. Barriers such as cost, time, can prevent the performance of waste management behavior, while altruistic factors, such as moral norms, may positively influence behavior despite the presence of barriers.

#### Agriculture

Only eight of the studies included in our review considered the relationship between behavioral determinants and agricultural environmental behavior. Multiple other studies, particularly from the disciple of rural research, were identified that examined the relationship between common psychological behavioral determinants and agricultural environmental behavior (e.g., Maybery et al., 2005; Minato et al., 2010). However, these studies did not meet our selection criteria, and many suffer from common errors (Burton, 2004), such as the misuse of scales and measures, the simplification of models, and the tendency to over-represent the attitude construct compared to other factors widely acknowledged in behavioral literature, such as social norms (Burton, 2004).

A common example of what Burton (2004) refers to as the, “failure of agricultural geography to develop theoretically” (p. 361) is the use of very broad measures of social factors, rather than measures that differentiate specific constructs, such as social norms (Burton, 2004). For example, Greiner and Gregg (2011) concluded that landholders are more motivated by stewardship values, and less motivated by “social considerations” on the basis of Likert scale responses to a single survey item that asked landholders to rate how motivated they are by being appreciated by society and colleagues.

Behavioral research that met our selection criteria indicates that factors such as attitudes (Wauters et al., 2010) and moral norms (Raymond et al., 2011) influence the adoption of conservation behavior by farmers. For example, Wauters et al. (2010) found that the decision to adopt soil erosion mitigation strategies, such as reducing tillage, planting cover crops, and planting buffer strips was explained by landholder attitudes.

A number of studies emphasize the importance of PBC and constraints for environmental agricultural behavior. For example, Lynne et al. (1995) found that PBC influenced both the initial decision of farmers to invest in water saving technology, and subsequent investment. Similarly, Borges et al. (2014) found that the adoption of strategies to improve natural grassland, including planting new species and using fertilizer, was influenced by attitudes, norms, PBC, and actual control. In particular, PBC was determined by knowledge, skills, and technical assistance, while actual control related to the cost of new technologies. In terms of constraints, (Keshavarz and Karami, 2016) found that farmers that experienced greater drought severity were less likely to adopt conservation behavior compared to those that experienced less drought severity.

Much less research has been conducted about the behavioral determinants of agricultural environmental behavior compared to common domestic behaviors such as recycling. However, the studies reviewed here suggest that agricultural behaviors are influenced by attitudes, PBC and constraints, particularly related to drought conditions, and the cost and skills required to adopt and maintain new behavior. In contrast to the other environmental behaviors included in this review, the studies considered here do not suggest that environmental agricultural behaviors are habitual.

#### Miscellaneous

Numerous other environmental behaviors have been considered in the behavioral literature, notably including multiple forms of political activism. However, many of these were excluded from our review because they did not meet the criteria specified. Two that did meet our criteria and do not fall into the above categories were visiting green hotels and signing a petition. Chen and Tung (2014) found that choosing to visit green hotels was related to the factors outlined in TPB and moral obligation. Hamid and Cheng (1995) tested a modified model of the TRA, incorporating past behavior and personal control. They found that signing an antipollution petition was related to past behavior and attitudes.

#### Summary of behavioral determinants

The findings of this review are consistent with the theoretical perspectives outlined above. Behaviors that are easier to perform are more likely to be explained by cognitive factors, while behaviors that are more difficult to perform, particularly those related to high costs, are more likely to be explained by practical factors, such as income. Few studies consider the maintenance of new behaviors. However, given that the initial experience with a new behavior is positive, and the ongoing activities involved are easy to perform, many pro-environmental behaviors that involve continued activities are likely to become habitual. Environmental behaviors related to transport, household consumption, purchasing, and waste management involve either a one-off cost, or ongoing behavior that is relatively easy to perform. By contrast, agricultural environmental behaviors are influenced to a greater degree by barriers and constraints, particularly in relation to drought. While the initial adoption of a new agricultural environmental behavior may be related to cognitive factors, over time the additional costs and effort associated with the changing activities involved for maintenance may result in the discontinuation of behaviors. Given the changing nature of agricultural environmental behaviors, and the influence of factors related to climate on the nature and amount of maintenance required, they are less likely to become habitual.

Fundamental to the success of environmental behaviors for mitigating problems like climate change is that new behaviors, such as reduced energy consumption and reduced car usage, persist over the long-term. For the purpose of designing effective interventions, policy makers should understand which behaviors are likely to be adopted and maintained relatively easily, compared to those behaviors that are likely to require additional incentives over the long-term. In the following we classify behaviors as one-off, continuous, or dynamic, depending on the activities involved for adoption and for maintenance, and discuss the implications for research and intervention.

### Classifying behaviors as one-off, continuous, and dynamic

Some behaviors involve a single activity, such as the purchasing of an energy efficient washing machine, or signing a petition. After the initial activity, no further cost or effort is required. These we term “one-off” behaviors. Many behaviors involve the frequent repetition of relatively easy and low-cost activities, such as recycling and reducing energy consumption in households. These we term “continuous” behaviors. Gardner and Stern (2008) make a similar distinction that specifically pertains to energy saving behaviors, between “curtailment actions” and “energy-boosting actions.” The former involves stopping an existing behavior while the latter usually involves substituting existing household products for energy efficient alternatives. They suggested,

“Curtailment actions must be repeated continuously over time to achieve their optimal effect, whereas efficiency-boosting actions, taking infrequently or only once, have lasting effects with little need for continuing attention and effort.” (Gardner and Stern, 2008, p. 17).

We build on this distinction in two ways. Firstly, our category “continuous” behaviors includes a range of curtailment actions, including reducing household consumption of energy and water. However, not all behaviors that involve the repetition of actions over time are related to curtailment. For example, recycling involves the repeated separation and disposal of recyclable waste, rather than curtailing the consumption of products that produce waste. Secondly, in addition to energy efficiency behaviors, our category of “one-off” behaviors includes actions related to water efficiency, in both domestic and agricultural contexts. Thus, while not dissimilar to the classification of energy-related behaviors outlined by Gardner and Stern (2008), our classification encompasses a greater variety of environmental behaviors.

Compared to one-off behaviors, continuous behaviors are repeated with some regularity. Those continuous behaviors that are repeated frequently are likely to become habitual. By comparison, other behaviors, particularly agricultural environmental behaviors, involve different activities for adoption compared to maintenance, and those activities may be performed less regularly. These we term “dynamic” behaviors. We argue that dynamic behaviors are likely to face more barriers to practice than either one-off or continuous behaviors, and are less likely to become habitual. Thus, dynamic behaviors pose the greatest challenge for policy makers.

Importantly, very little research considers whether environmental behaviors require maintenance, or what maintenance is involved for environmental behaviors to produce the intended outcomes. Table 1 summarizes what maintenance is likely to involve for common environmental behaviors to the best of our knowledge, and available information. In the following we outline the characteristics of one-off, continuous, and dynamic behaviors, present examples of each category, and put forward some recommendations for research.

#### One-off behaviors

Most behaviors that we define as “one-off” behaviors involve a single investment to replace an existing item with an environmentally friendly alternative, such as an energy efficient washing machine. The determinants of one-off behaviors vary depending on the nature of the activity involved. If a large cost is involved, or logistical barrier, such as the amount of effort required to access renewable technologies, psychological motivations likely to be unrelated to decision (Black et al., 1985; Steg, 2008). Examples of high-cost items from our review include household energy and water saving items, such as insulation, water conservation technologies for agricultural production, and renewable technologies. A number of other items also fall into this category, including water tanks. If a small cost is involved, such as for purchasing a small energy-efficient item (e.g., a kettle or lamp), cognitive factors may be more influential.

Switching energy companies from a traditional company to a green company also involves a financial investment. However, in this example the investment is for the fee associated with terminating a contract with the current company, and establishing a contract with the new company. Green energy is more expensive than traditional energy and therefore involves an elevated cost over the long-term. In the case of both purchasing expensive items, and switching energy companies, once the initial investment or effort has been made no further effort is required. The new item or contract is utilized in the same way as the old item or contract. The use of the new item, or contract falls into the same habitual pattern as the previous option (Bratt et al., 2015).

Some one-off behaviors involve a single activity that does not include a financial cost, such as signing a petition or attending an environmental rally. Signing a petition requires little effort and may be determined by cognitive factors (e.g., Hamid and Cheng, 1995). By contrast, the effort required and the determinants of attending an environmental rally are likely to vary depending on the nature of the rally and the context. Rallies that involve prolonged disruption to traffic and access to infrastructure, such as the anti-nuclear rallies in Melbourne, Australia during the 1990s to early 2000s often resulted in arrests (A. Dempsy, personal communication, March 11th, 2009). Anti-pollution rallies in China are known to result in violence (Lee and Ho, 2014). Therefore, the decision to participate is likely to be determined by different factors compared to the recent peaceful anti-climate change marches that have taken place in cities across democratic regions, including Australia (Cornish, 2017) and the United States (Cummings, 2017). Thus, while all one-off behaviors involve the performance of a single activity at a given point in time, the determinants vary.

#### Continuous behaviors

The behaviors that we define as “continuous” involve the same, or very similar, activities, and commitments for adoption and maintenance. Common examples of continuous behaviors include domestic recycling, water and energy saving, and choosing to take public transport to work rather than driving a car. Unlike one-off behaviors, many of the environmental behaviors we classify as continuous are relatively low-cost and low-effort to perform. This is because these behaviors involve the modification of pre-existing activities, and behavior modification is achieved more easily than the adoption of an entirely new behavior (Binder and Boldero, 2012). For example, most people brush their teeth regularly. Therefore, turning off the faucet while teeth brushing involves the modification of an existing behavior. However, some continuous behaviors do involve replacing an existing behavior with an entirely new behavior, and thus involve a moderate degree of effort. For example, switching from driving a car to using public transport is likely to require more effort than turning off the faucet while teeth brushing.

While the determinants of continuous behaviors vary, there are some common patterns. The initial adoption of a continuous behavior may be influenced by a range of factors related to altruism and self-determination, as well as practical factors. Behaviors that are easier to perform, such as purchasing products with eco-friendly packaging (Thøgersen, 1999) and domestic energy curtailment (Black et al., 1985), are more likely to be influenced by cognitive factors, while those that are more difficult to perform are more likely to be influenced by practical factors (Stern, 2000). For example, Davies et al. (2002) found that personal norms predicted curbside recycling behavior and argued that the, “task of putting household rubbish in a bin is a decision that employs a minimal amount of mental and physical effort on behalf of the householder.” (Davies et al., 2002, p. 31). By comparison, practical factors such as cost are more influential than cognitive factors when recycling involves transporting materials to a drop-off point and paying a fee (Guagnano et al., 1995).

With the exception of drop-off recycling, the continuous behaviors listed in Table 1 are performed with regularity, and therefore are likely to become habitual over time (e.g., Aarts and Dijksterhuis, 2000; Davies et al., 2002; Martínez-Espiñeira et al., 2014). Most energy and water curtailment behaviors occur daily, while choosing pro-environmental transport choices and eco-friendly purchasing are likely to occur weekly or daily. Curbside recycling involves separating waste on a daily basis, and putting a recycling container on the curb weekly or fortnightly. By contrast, drop-off recycling may occur with much less regularity, depending on household storage space and distance from a drop-off point. Reduced tillage was the only example of a continuous agricultural environmental behavior identified from our review, however there may be others that we are not aware of. Given the infrequency of planting seasons, it is possible that reduced tillage may become habitual only after years of repeating the new behavior.

The continued maintenance of environmental behaviors is rarely discussed in academic literature. However, studies suggest that while the determinants of the behaviors we term “continuous” vary, over time these behaviors are likely to become habitual. Thus, continuous behaviors should be maintained over the long-term. By contrast, dynamic behaviors present a much greater challenge for policy makers.

#### Dynamic behaviors

The behaviors that we term “dynamic” involve different activities for adoption and maintenance, therefore adoption and maintenance are likely to be influenced by different determinants. The examples considered below indicate that the amount of cost or effort required for maintenance is often greater than that require for the initial adoption of dynamic behaviors. Unlike continuous behaviors, maintenance is likely to be infrequent, or irregular, and may involve different activities from one instance to the next. Thus, dynamic behavior are less likely than continuous behaviors to become habitual.

Of the behaviors listed in Table 1, only those associated with agriculture, and purchasing expensive sustainable technologies that require ongoing maintenance, including switching to sustainable vehicles, are classified as dynamic. However, we are aware of others that are not addressed in our review, such as joining an activist group. Some forms of activism involve performing the same or similar activities with regularity, such as letter writing (Joireman et al., 2001). In other cases, the level of effort and the nature of activities vary considerably. Initially, joining an activist group is likely to involve an administrative process and attending group meetings. Over the long-term group members are called on to invest time and effort into the organization of direct actions, including rallies, education campaigns, and more elaborate and illegal protests. Organizations such as Greenpeace do not disclose the dates and activities involved for illegal direct actions, such as blockading an oil company, until close to the event (A. Dempsy, personal communication, March 11th, 2009). To participate group members may be required to take a day off work at short notice, or risk being arrested. The initial commitment of attending group meetings may well be related to cognitive factors such as attitudes toward the fossil fuel industry. The decision to engage in illegal activities at short-notice is likely to be related to numerous factors, including job security and legal record.

Dynamic behaviors that involve the purchase and continued use of sustainable technologies include switching to electric or hybrid vehicles. The initial adoption involves a high upfront cost, while maintenance involves the continued use of the purchased vehicle. The most common determinants of these behaviors are related to financial cost (e.g., Flamm and Agrawal, 2012; Hahnel et al., 2014; Lai et al., 2015; Barth et al., 2016). However, while the initial adoption of sustainable vehicles is higher than the cost of conventional vehicles, the continued use is generally less expensive than conventional vehicles (see section The Cost and Effort Involved for Adoption and Maintenance below). Thus, the upfront cost of purchase may deter adoption, while the long-term savings associated with use are a powerful driver both of adoption and continued maintenance (Ozaki and Sevastyanova, 2011; Graham-Rowe et al., 2012).

Agricultural environmental behaviors that we classify as dynamic include revegetation, either to provide a buffer between agricultural runoff and streams or to increase native species on riverbanks. Initially, revegetation involves planting seedlings. The maintenance of revegetation sites includes repairing damage from floods, storms, and animals, and extensive weeding (Moore and Rutherfurd, 2017). An additional example of dynamic agricultural behavior is excluding stock from riverbanks to improve water quality (Price and Leviston, 2014). Initially this involves constructing fencing along the riverbank, revegetation of the riverbank, and installing machinery for off-stream watering, such as troughs and pumps. Maintenance involves maintaining the revegetation site and repairing damage to infrastructure. For both revegetation and stock exclusion, the need for maintenance, and the nature of maintenance is influenced by factors that are unpredictable, such as flooding that promotes the growth of invasive weeds. Few studies have been conducted about the determinants of agricultural environmental behaviors that we term “dynamic,” and those that have only consider the initial adoption of new behavior. The adoption of these behaviors is influenced by factors related to both altruism (e.g., Raymond et al., 2011) and self-determination (e.g., Borges et al., 2014). However, rural research about landholder practices suggests that over time farmers struggle to maintain new conservation behaviors (Wilson et al., 2003, 2008).

In summary, agricultural environmental behaviors and switching to sustainable vehicles often involve different activities for adoption and maintenance, and therefore are dynamic. Sustainable vehicles are likely to be used regularly, and thus, the activities involved for maintenance may become habitual over time. By comparison, agricultural behaviors face unique barriers. Maintenance is less likely to become habitual because the activities required tend to be less routine, compared to other environmental behaviors such as recycling or choosing to take public transport rather than driving. Further, most environmental behavior research focuses on the performance of pro-environmental behavior in the daily lives of individuals, rather than behavior that effects their employment or income. In the agricultural context, environmental behavior is both performed in daily life by individuals, and is a component of landholder livelihoods. Environmental behavior tends to involve a shift from traditional agricultural practices, to practices that involve more time, effort, and cost. Therefore, these behaviors are susceptible to changing circumstances, such as drought or financial insecurity. For this reason, achieving permanent behavior changes, and thus permanent environmental improvements, may be more challenging for policy makers compared to relatively easy and low-cost domestic behaviors.

Below we consider the cost and effort involved for the adoption and maintenance of the environmental behaviors listed in Table 1.

### The cost and effort involved for adoption and maintenance

Understanding the cost and effort involved in the adoption and maintenance of a new pro-environmental behavior is essential for the design of interventions. Costs refer to the financial input required to perform a behavior, such as the cost of purchasing an electric car, or seedlings for revegetating a riverbank. Effort refers to any non-monetary input required to adopt and maintain a behavior, such as the time required to sort waste for recycling (Davies et al., 2002).

Table 1 demonstrates that most environmental behaviors can be classified into four categories according to the amount of cost and effort required for adoption and maintenance.

The first category includes behaviors that involve little, if any, cost for both adoption and maintenance. These behaviors require conscious effort to change established habits, such as taking shorter showers, turning the light switch off in an unused room, and curbside recycling.

The second category includes behaviors that involve a moderate cost and, or, effort for adoption, and for maintenance. These behaviors include drop-off recycling and purchasing organic produce. Drop-off recycling entails transporting waste to a site of disposal, and disposal often requires a fee. Purchasing fair trade produce involves higher costs than conventional produce, and accessing fair trade can be less convenient than accessing conventional markets (Tanner and Wölfing Kast, 2003). However, neither the cost nor effort required to adopt and continue performing these behaviors are exuberant.

The third category includes behaviors that involve an up-front cost for adoption and either no cost, or less cost than the conventional behavior for maintenance. Most behaviors that fall into this category involve a high initial cost, however in some cases the cost may be minimal, such as for purchasing a water efficient shower head or kitchen appliance. Maintenance can involve no additional costs, or less cost than the conventional alternative. Behaviors that involve a high initial cost and no additional costs include installing insulation and double glazing. Retrofitting a three bedroom house with double glazing or solid wall insulation can cost in the order of £4000, however no further expenditure is required over the long-term (Chapman et al., 2009). Behaviors that involve a high-cost initial cost for continued use include purchasing an electric car (Lipman and Delucchi, 2006) or an energy efficient refrigerator (Michel et al., 2015). In both cases the cost of purchasing sustainable technologies exceeds the cost of purchasing the conventional option, however, sustainable technologies consume less resources and therefore cost less than conventional options for use. For example, Granovskii et al. (2006) found that on average the purchase of electric cars retails for up to twice as much as conventional cars, while the cost of fuel for use is less than half the cost of conventional cars.

The fourth category includes behaviors that involve low or moderate costs for adoption and higher costs for maintenance. These behaviors are primarily agricultural, however there may be additional examples that we have overlooked. Examples include excluding stock from grazing riverbanks, and improving vegetation quality by revegetating degraded sites (Moore and Rutherfurd, 2017). Stock exclusion involves constructing low to moderately priced fencing for stock exclusion, depending on the length of fencing required. Fence repair and the provision of alternative watering sites for stock, including the use and maintenance of pumps and troughs, can exceed the effort and cost of fence construction. Similarly, the cost of adopting revegetation, including purchasing and planting seedlings, varies depending on the size of the revegetation site. However, over time the cost of maintenance is likely to exceed the initial cost of adoption; maintenance involves replacing seedlings that are lost to animal damage, flooding, or drought, and continual weed management that is both costly and time consuming (Ede and Hunt, 2008).

While Dembkowski and Hanmer-Lloyd (1994) suggest that cost and effort have a similar effect on the willingness of individuals to adopt environmental behaviors, such as making environmentally responsible consumer choices, we argue that the distinction is important from the perspective of designing interventions. In the following we consider the implications of our classification of environmental behaviors, in terms of the activities, costs, and effort involved for adoption and maintenance, for designing interventions.

## Designing environmental interventions

Policy makers often prefer to encourage individuals to voluntarily alter their behavior because promoting change through social interventions is perceived to be more popular within society, compared to legal regulation (Danne, 2003). To be effective, interventions to promote pro-environmental behavior should involve incentives that match the amount of effort and cost involved, both for the initial adoption, and long-term maintenance of the new behavior (Gunningham, 2003).

The idea that interventions should be designed according to the characteristics of individual behaviors is not unique (Lucas et al., 2008; Michie et al., 2008; Osbaldiston and Schott, 2012). Michie et al. (2011) suggested that interventions should be designed around the factors that influence the adoption of environmental behaviors. Osbaldiston and Schott (2012) observed that, “low-engagement treatments are appropriate for low-effort behaviors and that high-engagement treatments are effective for high-effort behaviors.” (p. 280). We support these views and add that in some instances multiple approaches may be necessary to firstly promote the initial adoption of a new behavior, and secondly ensure that the new behavior is maintained over the length of time required to be effective. Most environmental behaviors need to be maintained in perpetuity to mitigate environmental problems. We argue that the activities involved for maintenance, and the factors that are likely to influence maintenance, are equally important for the design of interventions, as the activities and factors related to adoption. The purpose of this paper is not to be prescriptive, however we can make some observations about the implications of our analysis and classification of behaviors for designing interventions, as follows.

One-off and continuous behaviors are less challenging for policy makers than dynamic behaviors. One-off behaviors involve a single effort or cost. Interventions that include financial subsidies are appropriate for promoting high-cost one-off behaviors (those classified as H/S in Table 1), such as installing insulation, and purchasing energy efficient white goods (Martínez-Espiñeira et al., 2014). Interventions that appeal to personal norms may be effective for encouraging the performance of one-off behaviors that involve a moderate cost or effort (those classified as M/M in Table 1), such as switching to a green energy company, and continuous behaviors, such as drop-off recycling (e.g., Guagnano et al., 1995; Bratt, 1999). Continuous behaviors often involve the repetition of relatively easy, low-cost activities that are likely to become habitual (those classified as L/L in Table 1) (e.g., Gilg and Barr, 2006). Existing habits, such as leaving the tap running while doing dishes, can be altered by changing the underlying goal of the behavior (Binder and Boldero, 2012) through interventions that involve education, social norms, and pro-environmental attitudes (Steg and Vlek, 2009). The water saving campaigns implemented by the Victorian Government in Australia during the last extended period of drought is an example of this approach. Studies of domestic behavior during and after the cessation of the water reduction campaign suggest that the emergence of social norms and pro-environmental attitudes resulted in new water saving habits (Walton and Hume, 2011; Binder and Boldero, 2012; Beal et al., 2014; Lowe et al., 2014). Habits can also be altered by changing environmental cues (Verplanken and Wood, 2006), such as by increasing access to public transport (e.g., Bamberg et al., 2003). Thus, policy makers can use interventions to break old habits, and form new habits that involve the repetition of activities that are low-cost and simple to perform.

Dynamic behaviors involve different activities, commitments, costs, and efforts for adoption and maintenance. Behaviors that involve a high initial cost and low ongoing costs, such as switching to a sustainable vehicle, may be encouraged through financial subsidies. For example, tax waivers have been found to increase the sale of hybrid cars by 10-fold (Gallagher and Muehlegger, 2011). Behaviors that involve a lower initial cost and higher ongoing costs and effort (those classified as H/L in Table 1) include agricultural behaviors aimed at improving water quality, such as planting native vegetation, growing run-off buffer strips, and excluding stock from waterways. While the adoption of new agricultural behavior is often related to cognitive factors, such as attitudes (e.g., Maybery et al., 2005), over the long term the cost and effort required for maintenance can become prohibitive (Wilson et al., 2003, 2008). In such cases it may be effective to use two separate interventions for adoption and maintenance. For example, an intervention that involves education is likely to be effective for promoting the adoption of revegetation. Over time, maintaining native revegetation sites can involve costly weed management (Curtis et al., 2008). A second intervention that involves subsidies for weed spray could be implemented to ensure that farmers maintain revegetation sites. Interventions to promote agricultural environmental behavior typically involve subsidizing the cost of adoption, such as constructing fences for stock exclusion, while landholders are responsible for maintenance (Moore and Rutherfurd, 2017). In the context of climate change, and associated financial insecurity (Horridge et al., 2005; Mpelasoka et al., 2008), this arrangement may poses a threat for the long-term success of agricultural environmental behaviors.

**Figure 1:**
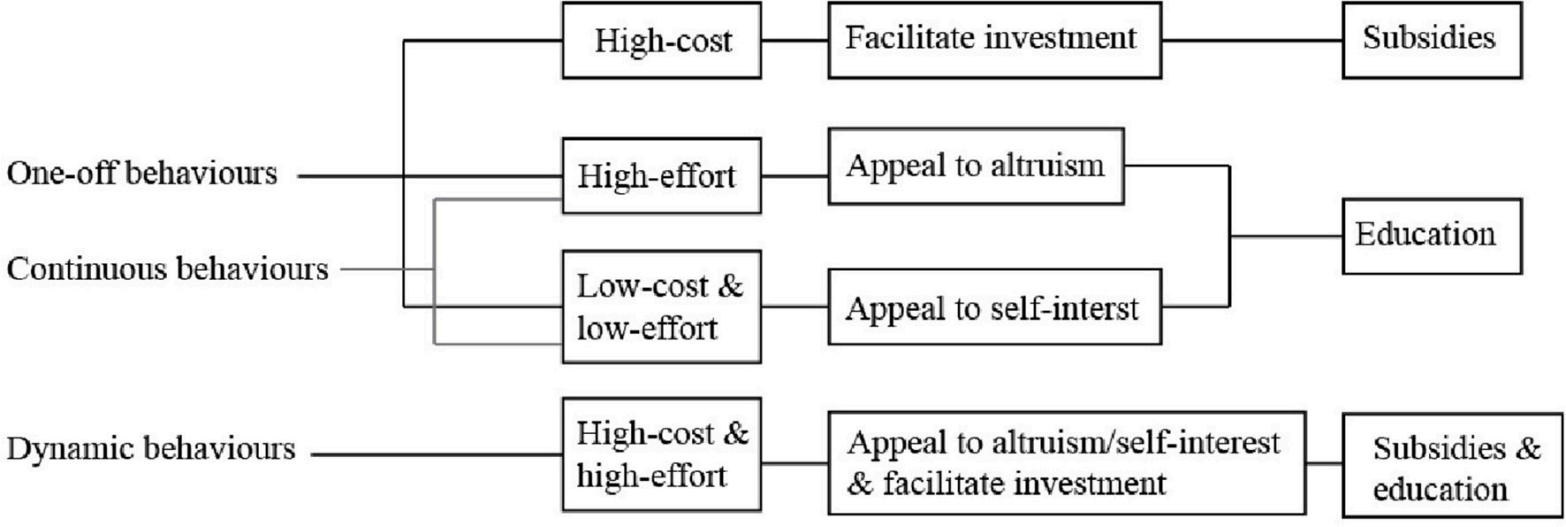
Flow-chart of cost, effort, and interventations for one-off, continuous, and dynamic environmental behaviors.

In summary, policy makers can choose from three main approaches to intervention that involve encouraging the voluntary adoption and maintenance of pro-environmental behavior: education campaigns that appeal to altruism or self-interest, the use of subsidies that facilitate investment in technology, or a combination of these two approaches (Danne, 2003; Gunningham, 2003). The flow chart presented in Figure 1 demonstrates how the barriers and determinants of behaviors can inform the design of interventions.

The distinction between continuous and dynamic behaviors is relevant for the design of interventions for a number of reasons. Firstly, behaviors that involve difficult activities, and face practical barriers will require greater incentives for adoption and maintenance than behaviors that involve activities that are easy to perform, and face few practical barriers (Steg and Vlek, 2009). Secondly, behaviors that involve reducing energy and water consumption are easier to promote than behaviors that involve changing energy sources and improving water quality. Changing energy sources, such as by installing solar panels, is expensive, while reducing energy consumption involves changing daily habits around transport, purchasing, and household patterns. Similarly, improving water quality involves dynamic agricultural behaviors that face substantial barriers, while reducing water consumption can be achieved by changing daily habits. It is fundamentally more difficult to replace existing behaviors with entirely new behaviors, than to make alterations to existing behaviors (Binder and Boldero, 2012).

## Conclusions

Understanding the characteristics of environmental behaviors is important for policy makers who wish to promote the voluntary adoption, and maintenance, of those behaviors in societies. To achieve ecological goals, many environmental behaviors must be continued indefinitely. We observe that some behaviors involve a one-off activity, while others require ongoing maintenance. Continuous behaviors are often low-cost, easy to perform, involve the same activities for adoption and maintenance, and are performed with regularity. Over time these behaviors are likely to become habitual, and thus will be maintained. Dynamic behaviors involve different activities for adoption and maintenance, and maintenance is performed irregularly. These behaviors are unlikely to become habitual, and may involve greater effort and cost to maintain than to adopt initially. This classification highlights the differences between those environmental behaviors that are performed in households and in everyday life on the one hand, and agricultural environmental behaviors related to the running of farm businesses on the other hand. Domestic behaviors such as recycling, and daily life behaviors such as purchasing and transport choices, are easier to perform and face fewer barriers. Agricultural behaviors are more difficult to perform and face significant barriers, such as drought and associated financial insecurity.

There are two main outcomes of this paper. Firstly, *researchers should choose theoretical approaches that are suitable for the target behaviors, including both the adoption and maintenance of behaviors*. In particular, research about the factors that influence both the adoption and maintenance of agricultural environmental behaviors is needed that adheres more rigorously to behavioral methodologies (Burton, 2004). Secondly, *policy makers should design interventions around the activities involved for the adoption, and maintenance of environmental behavior, and the factors that influence adoption and maintenance*. Behaviors that reduce consumption often involve the modification of existing behavioral patterns, such as reducing water use. Education campaigns that promote pro-environmental attitudes and social norms are effective for encouraging the adoption and maintenance of these behaviors. By contrast, behaviors that involve the performance of entirely new activities face greater barriers, such as switching to renewable energy and improving water quality, address the root cause of problems, such as climate change. Interventions that promote these behaviors are more likely to be effective if they include subsidies, and address the barriers to practice that emerge over the long-term, as well as for the initial adoption of new behaviors.

At the recent United Nations Framework Convention on Climate Change in Paris world leaders emphasized that to mitigate climate change societies must move beyond behaviors that are easily compatible with high-consumption lifestyles, such as using energy efficient lightbulbs. Limiting global warming to less than 2°C below pre-industrial levels will also require a substantial shift from fossil fuel consumption to renewable technologies (UNFCC, 2017). In the same vein, securing water resources for the future will require more than reducing domestic consumption. In drought prone Australia the agricultural sector is responsible for more than half of all water consumed annually (ABS, 2012). Stock grazing on riverbanks is a major contributor to the degradation of aquatic ecosystems and water quality worldwide (e.g., Jansen and Robertson, 2001; Agouridis et al., 2005). Policy makers face the challenge of promoting environmental behaviors that address the problems associated with climate change and water scarcity both at the source and the point of consumption. Designing effective interventions is essential for meeting that challenge.

## Author contributions

All authors listed have made a substantial, direct and intellectual contribution to the work, and approved it for publication.

### Conflict of interest statement

The authors declare that the research was conducted in the absence of any commercial or financial relationships that could be construed as a potential conflict of interest.
